# Targeting the Hippo Pathway and Cancer through the TEAD Family of Transcription Factors

**DOI:** 10.3390/cancers10030081

**Published:** 2018-03-20

**Authors:** Jeffrey K. Holden, Christian N. Cunningham

**Affiliations:** Department of Early Discovery Biochemistry, Genentech, Inc., South San Francisco, CA 94080, USA; holden.jeffrey@gene.com

**Keywords:** TEAD, Hippo, YAP, TAZ, cancer, transcription factor

## Abstract

The Hippo pathway is a critical transcriptional signaling pathway that regulates cell growth, proliferation and organ development. The transcriptional enhanced associate domain (TEAD) protein family consists of four paralogous transcription factors that function to modulate gene expression in response to the Hippo signaling pathway. Transcriptional activation of these proteins occurs upon binding to the co-activator YAP/TAZ whose entry into the nucleus is regulated by Lats1/2 kinase. In recent years, it has become apparent that the dysregulation and/or overexpression of Hippo pathway effectors is implicated in a wide range of cancers, including prostate, gastric and liver cancer. A large body of work has been dedicated to understanding the therapeutic potential of modulating the phosphorylation and localization of YAP/TAZ. However, YAP/TAZ are considered to be natively unfolded and may be intractable as drug targets. Therefore, TEAD proteins present themselves as an excellent therapeutic target for intervention of the Hippo pathway. This review summarizes the functional role of TEAD proteins in cancer and assesses the therapeutic potential of antagonizing TEAD function in vivo.

## 1. Introduction

**Figure 1:**
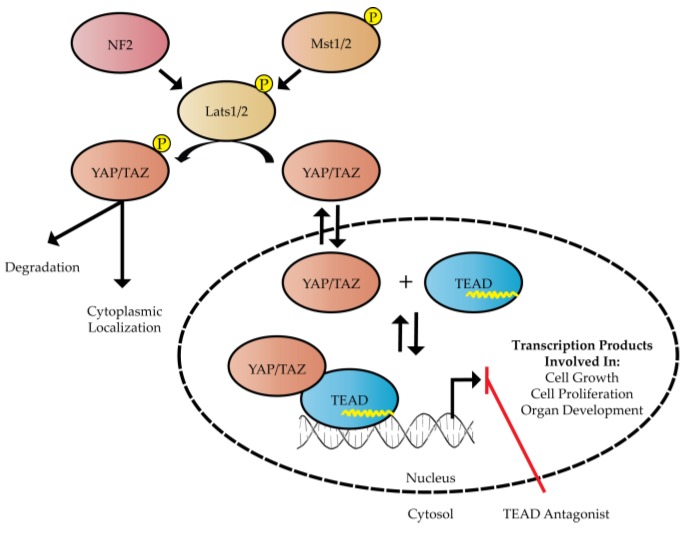
The Hippo signaling pathway is composed of neurofibromin 2 (NF2), mammalian STE20-like protein kinase 1/2 (Mst1/2) and large tumor suppressor 1/2 (Lats1/2). NF2, Mst1/2, and Lats1/2 are represented as red, orange, and brown ellipsoids, respectively. Phosphorylation of either Yes-associated protein (YAP) or transcriptional co-activator with PDZ-binding motif (TAZ) by Lats1/2 affects nuclear translocation of YAP/TAZ. Transcription of DNA can be activated by palmitoylated transcriptional enhanced associate domain (TEAD) in the presence of YAP/TAZ. The palmitate group is illustrated as a yellow line within the blue ellipsoid representing TEAD. A TEAD antagonist could serve as a useful strategy for altering the TEAD-YAP transcriptome in various cancer cells.

Transcriptional enhanced associate domain (TEAD) transcription factors function in response to the highly conserved Hippo signaling pathway to regulate cell growth and proliferation. Transcriptional activation by TEAD requires complex formation with a transcription co-activator and TEAD’s cognate DNA sequence. The known transcription co-activators that affect the function of TEAD include Yes-associated protein (YAP), transcriptional co-activator with PDZ-binding motif (TAZ, also known as WWTR1), Vestigial-like (VgLL) and p160 proteins [1]. However, we will mostly focus on YAP/TAZ as the main co-activators of the Hippo pathway in this review. The co-activator function of YAP/TAZ is regulated by the Hippo signaling pathway, which directs YAP/TAZ cellular localization via phosphorylation (Figure 1). When the upstream Hippo signaling pathway is turned on, large tumor suppressor 1/2 (Lats1/2) can be phosphorylated by mammalian STE20-like protein kinase 1/2 (Mst1/2) kinases or be activated by neurofibromin 2 (NF2), which serves as a scaffolding protein within the pathway [2]. Subsequently, phosphorylated Lats1/2 is able to phosphorylate YAP/TAZ in the cytoplasm. Importantly, phosphorylated YAP/TAZ is recognized by protein 14-3-3, which sequesters these co-activators to the cytoplasm and silences Hippo-induced gene transcription [3]. On the contrary, when YAP/TAZ are not phosphorylated, the co-activators are able to translocate into the nucleus by an unknown mechanism, bind TEAD transcription factors and activate transcription of Hippo target genes as shown in Figure 1. 

Current evidence indicates that the nuclear TEAD-YAP/TAZ complex is able to recruit the transcription enhancer, Mediator, and form a transcriptionally competent complex [4,5]. In humans, TEAD-YAP/TAZ driven transcriptional targets include genes involved in cell growth and proliferation, most notably connective tissue growth factor (CTGF) [6] and Cyr61 [7]. Recent ChIP-seq analyses has broadened our understanding of the transcriptional profile regulated by TEAD-YAP, which revealed that both proteins co-occupy >80% of promoters detected in MFC10A cells [6] and >75% of DNA sequences detected in breast cancer cells [8]. Zanconato et al. went on to demonstrate that oncogenic growth driven by TEAD-YAP co-localization is enhanced by transcription factor AP-1 [8]. In stark contrast, ChIP-seq analyses of two liver cancer lines revealed TEAD-YAP co-localization only occurred <7% of the time [4].

**Figure 2:**
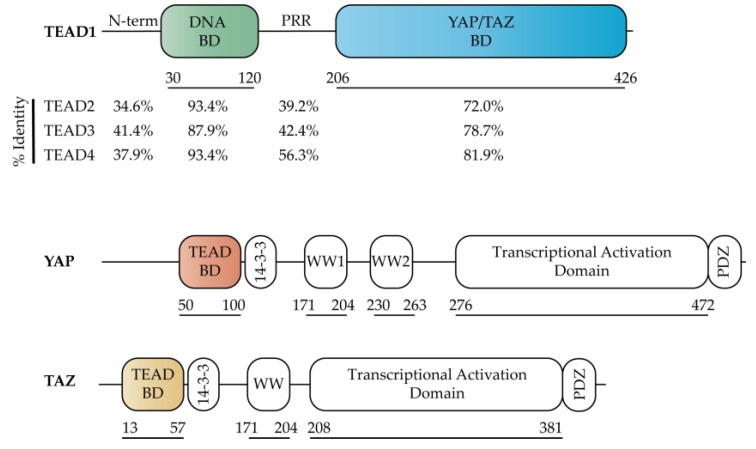
Domain organization of Hippo transcription factors TEAD, YAP (isoform YAP1-2γ), and TAZ. The percent identity for individual domains of TEAD1–4 was calculated relative to TEAD1 using Clustal Omega [13].

TEAD genes are a family of transcription factors that were originally identified through a genetic mosaic screen in *Drosophila* due to their critical role in organ development [9,10]. Unlike *Drosophila* that contains one TEAD gene, Scalloped, mammals have four TEAD genes (TEAD1, TEAD2, TEAD3 and TEAD4). TEAD1–4 are composed of a highly conserved TEA DNA binding domain (DBD) and YAP binding domain (YBD) [11,12], which is separated by a proline rich region (PRR) (Figure 2). Sequence alignments of the individual domains/regions indicate that the PRR and N-terminus account for the overall sequence differences as both DBD and YBD are very similar (Figure 2). While homology is high in both the DBD and YBD, these regions are not identical, with the DBD and YBD having an identity of 87.9% and 72.0% across all 4 members, respectively (Figure 2). 

Despite the high homology shared between human TEAD1–4, the individual TEAD proteins are differentially expressed in a tissue- and development-dependent manner. For example, TEAD1 is required for heart biogenesis [14], TEAD2 for embryonic development [15], TEAD4 for activating skeletal muscle genes [16] and TEAD3 has been shown to be specifically expressed in the placenta and several embryonic tissues during development [17]. Given the high degree of homology amongst these proteins, it remains to be seen if and how the individual TEAD paralogs complement one another in various physiological and oncogenic conditions and how their potential for synergism differentiates across tissue types.

In recent years, it has become well recognized that dysregulation of the Hippo pathway results in a cancerous phenotype. Aberrant Hippo signaling in mammals can be induced by single point mutations or altered expression levels of various Hippo pathway components [18]. Similarly, deactivation of the Hippo pathway results in cell growth and tumors [19]. Since TEAD is the downstream transcription factor of the Hippo pathway, inhibiting the function of TEAD is an attractive therapeutic strategy to reduce unwanted Hippo signaling and gene transcription. In this review, we focus on recent progress that has been made towards understanding the function of the TEAD family of transcription factors and discuss their utility as an oncogenic target for therapeutic intervention.

## 2. Structure and Function of Hippo Transcription Factors

Similar to the four TEAD paralogs, YAP and TAZ are also multi-domain proteins that are comprised of a TEAD binding domain (TBD), 14-3-3 binding region, 1–2 WW domains and a transcriptional activation domain (Figure 2). Binding of YAP/TAZ to TEAD in the nucleus is required to activate gene transcription, although the molecular mechanism by which this transcriptional activation occurs remains elusive at this time due to the lack of full-length TEAD structures in the presence or absence of a co-activator. Regardless, significant progress towards understanding the function and inhibitory potential of TEAD has been aided by structure-based studies focused on either the DBD or the YBD of TEAD. Specifically, structures of the YBD in the presence of co-activator TBD peptides [12] have provided critical information about the TEAD-YAP binding interface and form an excellent starting point for structure-based drug design of molecules that antagonize the TEAD-YAP complex. 

### 2.1. TEAD Co-Activators

The primary TEAD co-activators YAP and TAZ are composed of several domains that are connected by long disordered loop regions (Figure 2). Currently, only the TBD and WW domains of these co-activators have been structurally resolved [12,20,21]. The co-activator “activity” of YAP/TAZ is largely regulated by Lats1/2 phosphorylation, which generates a phospho-binding site on YAP/TAZ for the 14-3-3 protein [3], leading to its retention in the cytoplasm. Both YAP and TAZ also contain either 1 or 2 WW domains (Figure 2), which function to bind proline-rich motifs, such as the PPXY motif [22] (where X signifies any amino acid) present in Lats1/2. The C-terminal region of YAP and TAZ are composed of a transcriptional activation domain, which is partly composed of a coiled-coil motif. Of the 8 YAP isoforms identified, 6 isoforms have the coiled-coil motif disrupted [23]. The transcriptional activation domain has been shown to activate transcription when fused to Gal4 [24]. The C-terminal PDZ domain has also been implicated in TEAD transcriptional activation as disruption of the PDZ-binding motif significantly inhibited the oncogenic transformation of cultured cells when YAP was localized in the nucleus [25].

### 2.2. TEAD DNA Binding Domain (DBD)

**Figure 3:**
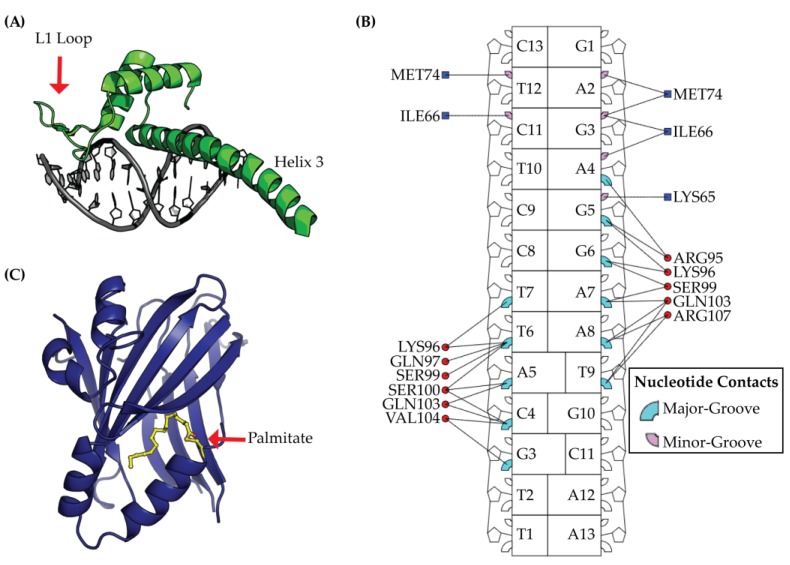
TEAD is a protein with multiple domains, which is composed of a DNA Binding Domain (DBD) and a YAP Binding Domain (YBD). (**A**) The DBD structure is illustrated from protein data bank ID (PDB) 5GZB and is composed of a homeodomain fold with three alpha helices (shown in green) bound to DNA (colored in grey); (**B**) Structural analysis of TEAD DBD using the interaction map from DNAproDB [34] illustrates the DNA major- and minor-groove protein residue contacts in cyan and pink, respectively. Nucleic acid contacts with DBD L1 loop are indicated by a blue square and contacts with DBD helix 3 are shown as red circles; (**C**) The YBD is post-translationally modified by palmitate (colored yellow) that extends towards the interior of YBD (colored in blue).

The TEAD DBD is composed of a helix-turn-helix homeodomain fold [26] that directs TEAD’s specificity towards DNA, which is shown in Figure 3. TEAD was first identified as a binder to the GT-IIc and SpH enhancers of the Simian Virus 40 and later as a binder to muscle-specific cytidine-adenosine-thymidine (MCAT) sequences [27]. With the advancement of sequencing technologies, such as ChIP-seq, our definition of TEAD-DNA binding has expanded to include additional binding sequences, which has been reported in previous studies [8,28]. Despite a broadened knowledge of TEAD-DNA interactions, the MCAT sequence of 5′-CATTCCT-3′ remains the predominant binding partner for TEAD [8]. This is not surprising considering that TEAD1–4 tightly binds MCAT sequences with a K_d_ range of 16–38 nM [11]. 

Transcription factor specificity to DNA is often increased by cooperative binding with specific co-factors [29]. Since multiple TEAD binding elements have been observed in promoters, such as CTGF [6], it seems feasible that TEAD cooperativity is biologically relevant and possibly necessary for the recruitment of transcription co-factors. Several groups have observed cooperative TEAD binding to DNA. However, cooperativity has only been observed in the absence of a co-factor [26,30,31,32]. In each of these studies, the DNA template was composed of two tandem MCAT binding sites. 

For understanding TEAD cooperativity, initial structure-function studies on TEAD1 indicated that the L1 loop is essential for cooperative binding to tandem MCAT DNA sequences [26]. Crystallization of a DBD-MCAT DNA complex revealed that L1 binds the minor groove of DNA (Figure 3) [33]. The crystal structure also revealed a second binding interface at the major groove with helix 3. Functional analysis of the DBD interactions at both the minor and major groove by mutagenesis revealed both sites to be critical for DBD function. Specifically, DBD mutants in these regions resulted in decreased TEAD promoter occupancy, decreased TEAD-DNA binding specificity, decreased YAP-induced transcription and decreased growth of gastric cancer cells [33]. Nonetheless, mechanistic details and the importance of TEAD cooperativity remain unclear and will need to be addressed by future studies.

### 2.3. TEAD YAP Binding Domain (YBD)

The TEAD YBD is composed of a β-sandwich fold with four α-helices [12,35] and is post-translationally modified by a palmitic acid [36,37] (Figure 3C). The palmitic acid is covalently attached to a conserved cysteine residue via a thioester bond and crystal structures of YBD reveal palmitoylation to bind a hydrophobic cavity located within the YBD β-sandwich fold [36]. Despite a structural understanding of YBD palmitoylation, the functional role and regulation of TEAD palmitoylation still remains unclear. Current evidence suggests TEAD palmitoylation to be essential for proper TEAD folding and cellular expression [38]. Immunofluorescence studies indicated that TEAD palmitoylation does not alter TEAD localization or trafficking, which has been observed for most other palmitoylated proteins in the cell [36,39]. TEAD palmitoylation may also be necessary for binding to YAP/TAZ [37], although this remains controversial as TEAD4 palmitoylation has been previously reported to not influence YAP/TAZ binding [38]. Given the role of palmitoylation on TEAD folding and stability, it is possible that this post-translational modification functions as an important regulatory checkpoint for TEAD protein synthesis and/or transcriptional activation. Unfortunately, evaluating the function of the palmitate group in vitro remains difficult due to the instability of mutants that lack the conserved cysteine. Based on the potentially important role of the palmitate, the lipid pocket is likely to be an important site for therapeutic intervention. 

**Figure 4:**
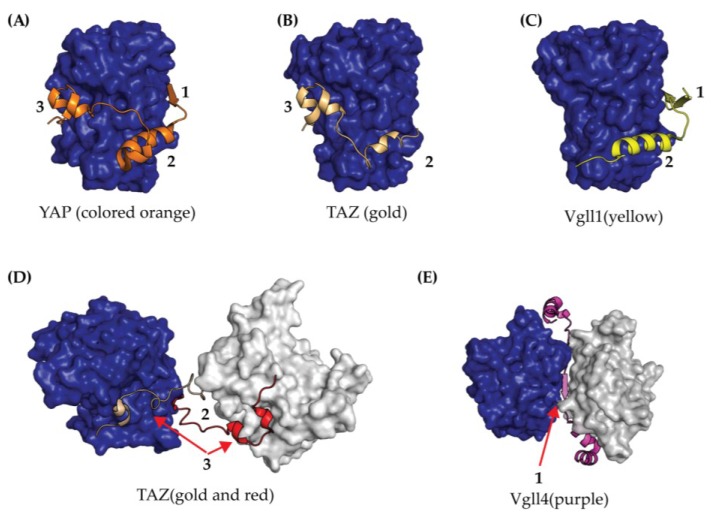
Crystal Structures of TEAD YBD (colored blue) bound to co-activator peptides from (**A**) YAP (orange); (**B**) TAZ binding mode one (gold); (**C**) Vgll1 (yellow); (**D**) TAZ binding mode two (gold and red); and (**E**) Vgll4 (purple). In both (**D**) and (**E**), the YBD is shown in blue and grey surface renderings since co-activators in the crystal structure induced dimerization. The TEAD binding interfaces for each co-crystal structure are labeled as 1, 2, and 3.

Crystal structures of the TEAD YBD forming a complex with the YAP-derived TBD peptide (amino acids 50–100) [12,40] have provided significant insights towards structure-guided approaches targeting the TEAD-YAP interface (Figure 4). In short, the YBD-TBD complex is composed of three interfaces. Interface 1 is composed of an anti-parallel β-sheet between the YBD and YAP residues 52–56. Interface 2 is stabilized by several hydrophobic interactions contributed by an α-helix from YAP residues 61–73. Interface 3 consists of both hydrogen bonds and hydrophobic interactions derived from a short loop spanning YAP residues 85–99, which is herein referred to as the Ω loop (Figure 4). Together, the interfaces 1–3 are highly conserved between TEAD1–4 [40] and stabilize binding to YAP with a K_d_ of 21 nM [38].

From mutagenesis and binding studies, the three TEAD-YAP interfaces have been extensively characterized with respect to one another. Initial cell-based studies demonstrated that mutations within the YAP Ω-loop negatively influence the formation of TEAD-YAP complex [12,35]. Recently, Mesrouze et al. further characterized the YAP Ω-loop and α-helix via mutagenesis [38]. The Ω-loop was found to serve as the primary energetic driving force at interface 3 for binding YAP with smaller, albeit important, energetic contributions from the YAP α-helix at interface 2 [38].

Biochemical studies indicate that the binding contributions from interface 1 are negligible [12] and it is possible that this interface is actually induced by the crystal packing as it is only observed in human TEAD1-YAP crystals (protein data bank ID (PDB) 3KYS) and not mouse TEAD4-YAP crystals (PDB 3JUA). This result is not very surprising as this region of YAP is known to be highly dynamic in solution [41] and only becomes ordered upon binding to TEAD [40]. Interestingly, structural comparisons of TEAD YBD in the presence (PDB 3KYS) and absence of YAP peptide (PDB 5EMV) revealed that YAP binding induces negligible changes to the YBD [42] with a root mean square deviation (RMSD) of 0.67 Å suggesting that YAP’s ability to activate TEAD transcription must come from additional interactions with other domains in the TEAD-YAP complex. 

The TEAD YBD has also been crystallized in complex with the Vgll1 peptide [43], Vgll4 peptide [44] and TAZ peptide [20], which is illustrated in Figure 4. Vgll1 was observed to bind to the interfaces 1 and 2 of TEAD [43] and Vgll4 adopted a different conformation by binding two YBD molecules [44] (Figure 4C,E). Thus, the binding modes of Vgll1 and Vgll4 are quite different than YAP/TAZ (Figure 4A,B) and may account for differences in transcriptional activation between the co-activators. The recent YBD-TAZ TBD crystal structure provided a new paradigm for TEAD biology as the TAZ TBD was observed in two distinct conformations [20]. In the first binding mode, TAZ TBD binds in a similar manner to YAP TBD, with non-covalent interactions between interfaces 2 and 3 (Figure 4B). In the second binding mode, one TAZ molecule binds two TEAD molecules to form a heterotetramer [20] (Figure 4D). Despite an incomplete structural view of full-length proteins, the new binding modes observed from a TAZ TBD peptide complexed to TEAD YBD might impact biological function and further explain differences between TAZ and YAP transcription profiles.

## 3. Physiological Roles of TEAD 

TEAD proteins are critical during early development and cellular senescence [14,45,46]. Although TEAD expression has been detected in almost all mammalian tissues, expression patterns of each TEAD paralog varies (detailed in Section 3.1). In mammals, TEAD1–4 are differentially expressed in a cellular- and development-dependent manner. Hence, it is likely that each TEAD has either a context-dependent function or that TEAD subsets operate together. In recent years, it has also become well recognized that specific TEAD proteins play an important role in cancer as activators of pro-growth gene transcription. 

### 3.1. TEAD in Early Development

The physiological function of TEAD genes in early development was first characterized in animal models when mice lacking the TEAD1 gene failed to develop a proper heart and died at embryonic day 11–12 [14]. Strikingly, unlike TEAD1 knockout mice, TEAD2 knockout mice appeared normal, although they had an increased risk of exencephaly [45,46]. The embryonic lethality observed in TEAD1 knockout mice was exacerbated in TEAD1/2 double-knockout mice as mice died at embryonic day 9.5 [45], suggesting that TEAD1 and TEAD2 compensate for one another. Knockout of TEAD4 is also lethal as the mice embryos fail to establish a trophectoderm cell lineage [47]. However, if TEAD4 is disrupted by Cre recombinase after embryonic day 6.5, the embryos complete development, which suggests that TEAD4 is uniquely responsible for establishing the trophectoderm cell lineage in early development [47]. Despite differences observed in early development for TEAD1, 2 and 4 a TEAD3-deficient mouse has yet to be described; hence, the exact function of TEAD3 during development is currently unknown. Recently, one study in mice provided evidence that TEAD3 plays an important role in DNA methylation [48]. However, follow-up studies to support this observation have yet to be reported.

### 3.2. Oncogenic Function of TEAD

Multiple tumor types overexpress TEAD genes [49,50,51,52,53,54,55,56] and this overexpression is well established as being directly correlated to the expression of pro-growth factors, such as CTGF [6], Cyr61 [7], receptor tyrosine kinase AXL [57], Myc and survivin [58]. TEAD has also been shown to upregulate the mesothelin gene, a well-established tumor marker [59]. In prostate cancer [55], gastric cancer [50] and colorectal cancer [52], increased TEAD levels serve as a useful prognostic marker as TEAD expression levels correlate with poor clinical outcomes. In sharp contrast, TEAD expression can also be downregulated in some cancers, including renal and various breast cancers [1]. Given the importance of TEAD expression and the genes regulated by TEAD activity, TEAD proteins are thus likely important mediators of tumorigenesis and attractive therapeutic targets. 

### 3.3. TEAD is Required for YAP/TAZ Oncogenesis

YAP and TAZ are well defined oncogenes that are overexpressed in many cancers [6,60]. In hepatocellular carcinomas, YAP serves as a prognostic marker for poor survival rates [61]. Overexpression of YAP/TAZ leads to an excess of co-activator proteins that are not phosphorylated by upstream Hippo kinases and thus, are able to enter the nucleus and activate TEAD transcriptional activity. Association of YAP/TAZ with TEAD is critical as mutations in the TBD-YBD interface alter transcription profiles due to poor complex formation and subsequent insufficient co-activation [6]. Nuclear co-localization of TEAD and YAP is best evidenced from ChIP-seq experiments, which observed both transcription factors to co-localize on DNA at enhancer sites that contact their regulated promoters through DNA looping [8]. In addition, it is well established that TEAD is required for YAP oncogenesis in cell culture [6]. Altogether these data indicate that YAP oncogenesis is most likely TEAD-dependent.

### 3.4. TEAD Modulates YAP Oncogenesis 

In a landmark study, Liu-Chittenden et al. demonstrated that YAP-induced proliferation can be suppressed by expression of a mutant TEAD2 that lacks the DNA binding domain (TEAD2Δ1-111) [62]. Overexpression of TEAD2Δ1-111 led to a dominant-negative phenotype that suppressed tumorigenesis in the liver, inhibited YAP’s antiapoptotic function and maintained normal liver growth [62]. Similarly, overexpression of full-length TEAD1 by transfection to mouse 3T3 cells was found to lower YAP activity in a dose-dependent manner [63]*,* resulting in a dominant negative phenotype. A dominant negative phenotype has also been observed in vitro using gastric cancer cells HGC-27 by transfecting TEAD4 mutants that disrupt DNA binding [33] and in *Drosophila* by co-expression of a truncated Scalloped gene [64]. Moreover, transgenic mice engineered to express TEAD1-Y421H, a missense mutation at interface 3 that limits TEAD-YAP complex formation and results in the human disease Sveinsson’s chorioretinal atrophy (SCRA; also referred to as helicoid peripapillary chorioretinal degeneration, atropia areata or circumpapillary dysgenesis), was found to shrink xenograft tumors via a dominant negative mechanism [65]. Finally, a dominant negative mechanism has been reported in cancer patients with elevated levels of isoform TEAD4-S (aberrant splicing isoform that results in deletion of the TEAD4 DBD) as these patients have an increased rate of survival [66]. Together, these data indicate several important observations about TEAD. First, cellular levels of TEAD are critical. Second, YAP oncogenesis due to YAP overexpression and nuclear localization can be turned off by increasing TEAD protein levels. Finally, therapeutically targeting TEAD to block the TEAD-YAP interaction is a promising strategy for YAP-dependent tumors. Thus, an improved mechanistic understanding of full-length TEAD is critical towards the development of novel inhibitors that modulate TEAD transcription.

## 4. TEAD as a Therapeutic Target

As our understanding of Hippo pathway biology and regulation advances, TEAD proteins have emerged as promising therapeutic targets for antagonizing Hippo transcription under oncogenic conditions. Unlike YAP/TAZ, TEAD1–4 are mostly composed of structured domains. Development of a TEAD antagonist is likely to be a challenging task, as the inhibitor will need to localize to the nucleus and bind TEAD with high affinity and specificity. However, unlike a traditional enzyme with a single active site, there are several potential vectors for therapeutically antagonizing TEAD. Due to the high conservation between TEAD1–4 and the lack of detailed knowledge for each paralog’s functional role, it remains to be understood if an inhibitor will need to be specific for one member of the family or pan-specific for all human TEAD proteins. 

### 4.1. Targeting the DNA Binding Domain (DBD)

Selective targeting of the TEAD DBD has yet to be reported and no inhibitors are currently available. As a first proof of concept for targeting the DBD, site-directed mutagenesis on residues that bind the minor and major groove of DNA (Figure 3B) were found to slow tumor cell growth [33]. Based on binding studies and a crystal structure, the mutated residues that slowed tumor growth were at positions critical for binding to the minor and major groove of the MCAT DNA sequence [33]. These regions have also been proposed as two of three potentially sites of therapeutic targets on the DBD [67]. Due to the high sequence conservation of the DBD (Figure 2), inhibitors that target this region would be pan-specific and provide a useful tool for further understanding TEAD biology. However, targeting transcription factors through their DNA-binding domains has been traditionally quite difficult. Interactions between proteins and DNA are usually mediated by alpha-helices, which reach into DNA grooves with their side chains to create sequence-specific interactions. This type of interface is usually devoid of small pockets that typically lend themselves to be conducive for small molecule ligand discovery. Additionally, the highly-charged nature of the protein-DNA interaction would indicate that for a small molecule to disrupt this interaction, it also would likely need to be charged and thus, limit the required membrane permeability properties to engage TEAD in the nucleus.

### 4.2. Targeting the Lipid Pocket

**Figure 5:**
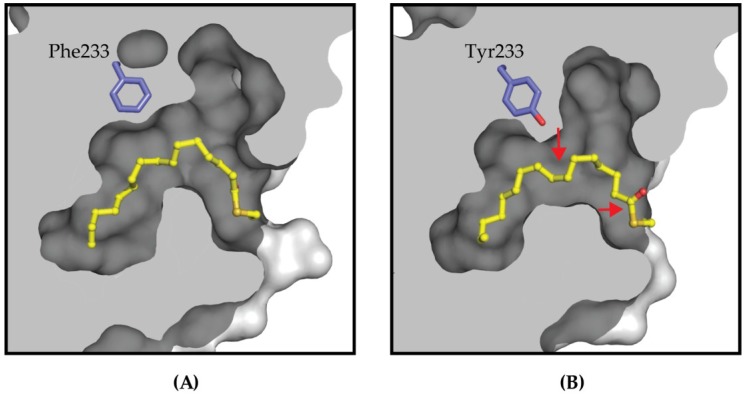
The palmitate group (colored yellow) extends towards the interior of YBD shown as a grey surface representation. The lipid pockets of (**A**) TEAD2 and (**B**) TEAD3 are identical except for the Phe/Tyr difference, which is shown in blue stick format. For TEAD3, the bulkier Tyr233 requires the lipid chain to reorient relative to the TEAD2 structure. The most noticeable reorientations are marked with red arrows on the TEAD3 lipid pocket to illustrate the alkyl chain moving down and the carbonyl group rotating out towards the solvent.

The YBD is stabilized by a post-translational palmitoylation at a conserved cysteine [36] that extends into a hydrophobic cavity within the domain (Figure 5). The conservation of this pocket is quite high amongst the four TEAD paralogs, but they are not identical. This indicates that discovering pan-TEAD specific molecules against this pocket may be problematic. The first evidence to support this comes from the TEAD3 structure at Tyr233 where all other TEAD paralogs have a Phe residue. The subtle difference of Tyr compared to Phe, due to a hydroxyl group, changes the volume and hydrophobicity of the lipid pocket, resulting in the lipid shifting downward in the pocket (Figure 5A). The steric hindrance and chemical properties of the hydroxyl group present on this Tyr in TEAD3 may also prevent chemical matter from binding TEAD3 in the same mode as the other TEAD paralogs and thereby, prevent pan-TEAD specificity.

However, unlike the DBD, chemical matter has been reported to bind the TEAD2 and TEAD4 lipid pocket and alter Hippo pathway biology. The first indication of the lipid pocket as a therapeutic target was from a high-throughput screen using dynamic scanning fluorimetry to identify ligands that stabilize the TEAD4 YBD [68]. From this initial screen, flufenamic acid and analogs were found to bind the lipid pocket (flufenamic acid K_d_ = 73 μM) and lower transcription levels of gene products driven by TEAD-YAP [68]. While molecules that target the lipid site have been shown to exert biological changes in the TEAD transcriptome, a mechanism of action remains unclear as the crystal structure of flufenamic acid forming a complex with TEAD2 YBD (PDB 5DQ8) was unchanged relative to the native structure (PDB 5EMV) with an RMSD of 0.37 Å. 

Additionally, understanding the potency requirements to obtain biological effects remains to be understood as displacing a covalently bound ligand will be challenging in vivo*.* Future work that targets the lipid site will first need to address the mechanistic and regulatory functions of this post-translational modification before it can be fully understood how TEAD function can potentially be modulated with any therapeutic benefit by a lipid pocket antagonist.

### 4.3. Targeting the TEAD-YAP Interface

Disruption of the TEAD-YAP interface is currently the most promising therapeutic strategy for augmenting transcriptional outputs of the Hippo pathway. Both biochemical and structural studies demonstrate that the primary TEAD-YAP interaction occurs between three highly conserved YBD interfaces and YAP residues 50–100 [40], with interface 3 determined to be the most critical for YAP binding [38]. One of the first small molecule screens reported to identify binders of this interface utilized a library of previously approved FDA molecules. This screen identified the porphyrin, Verteporfin, as a top hit, which is currently prescribed as a photosensitizer for macular degeneration [69]. However, upon functional characterization, Verteporfin was found not to act directly with TEAD but rather to function as a competitor for binding to YAP, which was observed by its co-elution with YAP during chromatography studies [62]. The exact mechanism by which Verteporfin is able to affect Hippo signaling remains an active area of investigation by many groups. Recently, another small molecule campaign also identified a fragment that binds to interface 2 of TEAD with >300 μM K_d_ [70]. Based on molecular dynamics studies, the authors speculate that the initial hit can be further optimized by building the fragment into a nearby cryptic binding site to improve binding affinity [70]. Further work will be required to validate this site and fragment as a useful therapeutic target and TEAD antagonist, respectively.

One challenge with targeting protein–protein interactions, such as TEAD-YAP, is that the native surface contacts between protein–protein interfaces can be extensive and relatively featureless. This makes the discovery of small molecule ligands that antagonize these interfaces generally difficult to identify and usually requires potent binding to a smaller “hot-spot” in order to modulate the interface [71]. Fortunately, two therapeutic hot spots at TEAD-YAP interfaces have been identified and might be used to overcome this obstacle [38]. The hotspots are centered on YAP residues F69 and R89 [38]. The role of these residues and their interactions with TEAD will need careful consideration for future development of TEAD inhibitors. 

Given the aforementioned difficulty to identify small molecule TEAD-YAP interface binders, there has been significant efforts exerted for generating larger peptide-based therapeutics to antagonize the TEAD-YAP/TAZ interface. One promising therapeutic strategy reported was the rationally designed cyclized YAP Ω loop peptide mimetics. Through the use of structural biology and modeling, Zhang et al. were able to convert YAP residues 84–100 with an IC_50_ of 37 μM into a disulfide stapled macrocycle with an IC_50_ of 25 nM. In addition, the engineered macrocycle was functional as it displaced the YAP amino acid construct made of the residues 50–171 from TEAD [65]. A separate study also utilized computational modeling and a mammalian display platform to identify cystine-dense peptides (CDP) that inhibited the TEAD-YAP interaction [72]. Based on the computational approaches, Crook et al. were able to design an initial library and identify a CDP that targeted interface 2 of the TEAD-YAP interface. The designed CDP was demonstrated to inhibit TEAD-YAP interaction via co-immunoprecipitation and bind tightly with a K_d_ of 31 nM [72]. From the mammalian display platform, the TEAD CDP identified was shown to function as a proteolytically and thermally stable peptide, although this did not demonstrate any effect in cell-based assays presumably due to poor membrane permeable properties.

Another consideration for the design of inhibitors that target the TEAD-YAP interface is that these molecules could also antagonize the function of co-activators Vgll and p160. Since Vgll1 and Vgll4 bind to interfaces 1 and 2 of YBD but not 3, an inhibitor that binds to interface 3 may not influence the activity of Vgll1 and Vgll4. However, the benefit of selectively targeting one co-activator over another remains to be seen and significant work is required to better understand the role of TEAD co-activators, such as Vgll and p160 proteins, with respect to canonical Hippo signaling.

Early efforts to perturb the TEAD-YAP interface have provided promising chemical matter: both small molecules and peptides. For purposes of developing a drug, significant work in this area will be required to generate a cell permeable and specific TEAD inhibitor. Moreover, while targeting a protein–protein interface remains a challenge regardless of the target, the fact that the TEAD-YAP/TAZ binding interface is identical amongst all TEAD paralogs indicates that a pan-TEAD selective compound is possible. Development of a TEAD-YAP interface binder will require an advanced understanding of the proper biomarkers for monitoring during treatment of Hippo-dependent tumors and a careful evaluation of TEAD antagonists in healthy cell lines.

## 5. Challenges and Future Perspectives

In recent years, the Hippo signaling pathway has emerged as a promising onco-therapeutic target due to its critical role in promoting cell growth and cell survival. Recognition of TEAD and YAP as bona fide oncoproteins has led to a significant effort to understand their therapeutic potential. However, despite the substantial amount of biochemical, structural and biological characterizations that have taken place since the discovery of this pathway, critical questions remain unanswered and will provide several arenas for future research.

One of the most exciting questions to address is identifying the optimal therapeutic target for modulating Hippo signaling in cancerous cells. Certainly, TEAD has emerged as a promising target since YAP/TAZ are mostly unstructured and inhibition of the Hippo kinases would be deleterious, as Lats1/2 and Mst1/2 function as tumor suppressors. Further characterization of TEAD as a therapeutic target will require the generation of new tool compounds to evaluate the role of TEAD in cancerous and healthy cells. Specifically, development of compounds that target the TEAD lipid pocket will aid in understanding the essential role of this post-translational modification and potentially uncover its therapeutic relevance. This will also require understanding whether TEAD palmitoylation is spontaneous or regulated by an acyl-transferase. If it is the latter, an acyl-transferase could serve as a new vector for potentially modulating Hippo pathway transcriptional output.

A mechanistic framework for various aspects of the Hippo pathway remain unclear, especially pathway activation/deactivation and transcriptional activation. First, we do not fully understand how extracellular cues can turn the Hippo pathway on/off. At the other end of the pathway, we still have an incomplete understanding of how the TEAD-YAP complex activates transcription structurally and biochemically. Specifically, we lack a full understanding of how TEAD-YAP-DNA associate and dissociate in the nucleus. It is possible that the TEAD-YAP complex is disrupted in the nucleus by a post-translational modification, a nuclear signal or re-localization of proteins. Certainly, an improved mechanistic framework of TEAD-YAP association and dissociation with DNA will provide novel insights towards the development of new therapeutics.

Finally, and most clinically relevant to TEAD, it is important to investigate whether an inhibitor that turns off TEAD-YAP transcription in tumors can be efficacious and not deleterious in healthy cells. Furthermore, a related question involves investigation to determine at what stage of development the Hippo pathway can be targeted. Early evidence in liver cancer models, a YAP driven cancer, suggest that overexpressing TEAD does not impair the development of normal tissue [62]. Therefore, it seems feasible to develop efficacious inhibitors that stabilize the balance of the TEAD-YAP complex within the nucleus. However, this remains to be fully understood without a potent inhibitor that selectively targets the TEAD-YAP complex and more studies will be required. Equally important future work will require identifying and characterizing biomarkers for Hippo-dependent tumors. Currently, there is a large disparity in understanding the transcription profile in various tumors as TEAD-YAP co-localization varies significantly [4,8]. Certainly, a broad examination of tumor types will deconvolute the complexity of TEAD transcription profiles as we also gain an improved understanding of TEAD1–4 function induced by various transcription factors. Therefore, elucidation of Hippo biomarkers will be a key step for evaluating the efficacy of inhibitors that target the Hippo pathway and is one of many challenges that need to be overcome before transitioning a TEAD inhibitor into the clinic. 

Over the past decade, significant strides have been made towards the characterization of therapeutic targets within the Hippo pathway. Although development of Hippo inhibitors remains in its infancy, advancement and identification of new tool compounds from chemical screens and display platforms will continue to expand our understanding of Hippo signaling components. Continued progress in this field is likely to provide critical insights for cancer biology and perhaps a novel therapeutic option for the treatment of various cancers.
